# Optimal period for achieving sustained unresponsiveness in peanut oral immunotherapy

**DOI:** 10.5415/apallergy.0000000000000110

**Published:** 2023-09-07

**Authors:** Kosei Yamashita, Toshinori Nakamura, Takanori Imai, Aiko Honda, Yuki Okada, Mayu Maeda, Taro Kamiya

**Affiliations:** 1 Department of Pediatrics, Showa University School of Medicine, Tokyo, Japan

**Keywords:** Food allergy, oral food challenge, oral immunotherapy, peanut, sustained unresponsiveness

## Abstract

**Background:**

Oral immunotherapy (OIT) can help children with persistent food allergies achieve sustained unresponsiveness (SU). However, the optimal therapeutic period for obtaining SU remains unclear.

**Objective:**

We aimed to retrospectively investigate the association between the OIT treatment period and achievement of SU.

**Methods:**

We enrolled patients who received OIT for peanut allergy between January 1, 2018 and December 31, 2022. OIT comprised the build-up phase, maintenance phase, complete avoidance, and an oral food challenge (OFC) for confirming SU. The peanut dose in the OFC was gradually increased to 3,000 mg (peanut protein: 795 mg), which was subsequently maintained for ≥5 months. SU was defined as a negative response to 795 mg of peanut protein after ≥2 weeks of complete avoidance. We evaluated the therapeutic OIT period for achieving SU using Kaplan–Meier analysis.

**Results:**

Forty-eight patients underwent peanut OIT. The starting age at OIT initiation was 8 (interquartile range [IQR], 7–10) years. Forty-one (85%) patients had a history of anaphylaxis. The median specific immunoglobulin E concentration to peanut and Ara h 2 at OIT initiation was 85.3 (IQR, 33.7–100) and 57.6 (IQR, 21.9–100) UA/mL, respectively. The median observational period was 2.1 (IQR, 1.6–3.0) person-years (PY). Thirty-four (71%) patients achieved SU, with the rate of SU achievement gradually increasing with the therapeutic period. The median period until SU achievement was 2.1 (95% confidence interval, 1.6–2.5) PY. The rate of SU achievement slowed down after 2.7 PY.

**Conclusion:**

OIT for at least 2.7 PY can increase the rate of SU achievement. The protocol No. 3107.

## 1. Introduction

Peanuts are a major food allergen and account for 5% of all cases of food allergy (FA) in Japan [1]. Allergies to egg, milk, and wheat generally resolve during childhood [2-4]; however, peanut allergy is likely to persist throughout life, resulting in chronic fear of accidental ingestion and anaphylaxis among patients [5]. Accidental exposure to peanuts is a common occurrence, with an annual incidence of 12% to 15% among children with peanut allergy [6, 7]. The standard of care is allergen avoidance and rescue medication in case of allergic reactions or anaphylaxis [8, 9]. Immunotherapy could help modify the prognosis of peanut allergy to improve the quality of life (QOL) of patients.

Oral immunotherapy (OIT) has recently become a promising treatment for patients with immunoglobulin E (IgE)-mediated FA [10, 11]. It is an experimental treatment for persistent FA (Japanese guidelines on FA do not recommend OIT as a general practice among patients with FA) [1]. It involves ingestion of the allergenic food, with the ingested amount being gradually increased to induce desensitization [12]. Given the lack of other treatment options for peanut allergies, the therapeutic potential of OIT for peanut allergy is garnering increasing interest [10]. Previous studies on peanut OIT have demonstrated that it can increase the individual doses required to provoke allergic reactions, leading to desensitization [13-16] or sustained unresponsiveness (SU) [13, 15, 17, 18]. SU is defined as the ability to safely consume a serving of food containing the trigger allergen for a period of time after stopping allergen immunotherapy [19]. A systematic review demonstrated that peanut OIT for a median period of 31 weeks was required to achieve desensitization [20]. However, the optimal therapeutic period for achieving SU remains unclear.

Some patients respond poorly to peanut OIT and fail to achieve SU within the study periods. A recent study on OIT for hen eggs reported that a longer duration of OIT can lead to a higher rate of SU achievement [21]. Accordingly, we usually extend the period of peanut OIT for patients who fail to achieve SU within several years. However, the extent to which a longer period of peanut OIT increases the rate of SU achievement remains unclear. Since OIT may provoke allergic reactions during treatment [22, 23], it is important to determine the optimal therapeutic period to help patients decide whether to continue therapy.

This study aimed to investigate the association between the OIT period and SU achievement as well as to clarify risk factors for a poor response to peanut OIT at initiation.

## 2. Materials and methods

### 2.1. Study design, population, and outcome definition

We included patients with an IgE-mediated FA to peanuts who underwent OIT between January 1, 2018 and December 31, 2022 at the Department of Pediatrics at Showa University Hospital, Tokyo, Japan. We included patients aged 6 to 15 years at the start of OIT who were followed up for >280 days from OIT initiation. Peanut allergy was confirmed using either a positive result of open oral food challenge (OFC) within the previous 6 months or a history of an obvious allergic reaction within the previous 6 months. Peanut sensitization was defined as specific IgE levels ≥0.7 UA/mL in reaction to peanut or Ara h 2 intake within the last 6 months. All participants provided written informed consent for the study before OIT initiation. OIT was not administered to participants with a history of peanut-induced anaphylactic shock, uncontrolled bronchial asthma or atopic dermatitis (AD), or respiratory or cardiovascular medical complications. This study was approved by the Medical Ethics Committee of Showa University (SHOWA University Clinical Research Review Board, 1-5-8, Hatanodai, Shinagawa-Ku, Tokyo, 142-8666, Japan. The protocol No. 3107. The Chairperson name of the ethics committee is Masahiko Izumizaki. The date of approval in April 14th, 2020.).

### 2.2. Primary outcome

The primary outcome was the treatment period until the achievement of SU to peanuts.

### 2.3. Secondary outcome

The secondary outcomes were the period until the achievement of desensitization, frequencies of adverse events and adrenaline intramuscular injections during OIT, changes in peanut-specific and Ara h 2-specific IgE, and risk factors for failure to achieve SU.

### 2.4. OIT protocol

**Figure 1.**
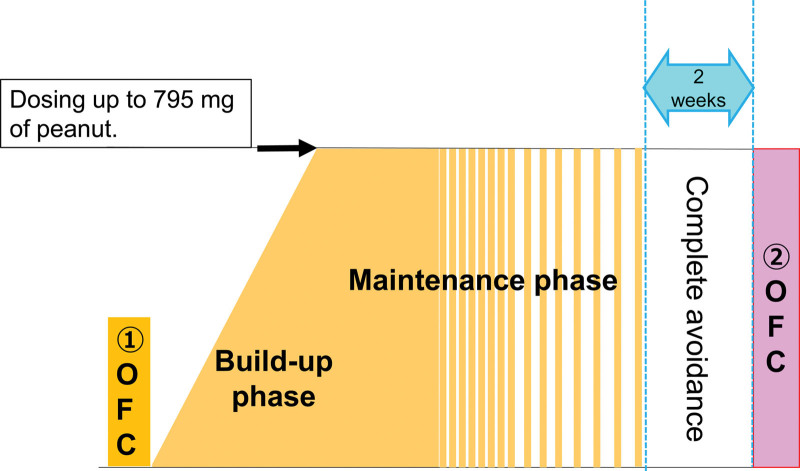
OIT protocol. OFC, oral food challenge; OIT, oral immunotherapy.

Figure 1 and Supplementary Table 1 http://links.lww.com/PA9/A6 show the details of the OIT protocol. The protocol comprised the initial OFC, build-up phase, maintenance phase, complete avoidance, and final OFC for confirming SU.

To determine the initial daily dose, patients underwent an initial OFC at the start of the study. The amount of peanuts consumed at the initial OFC was determined by the attending physician based on the patient’s medical history. The peanuts were weighed and prepared by the family and confirmed by the physicians before OFC initiation. In the OIT, the patients began by consuming lower amount of peanut if the OFC positive; if the OFC was negative, this dose was adopted as the starting dose of OIT, which varied across patients. However, the subsequent OIT protocol was similar for all patients. Patients were instructed to limit activity for 1 hour after ingesting peanuts. In the build-up phase, the patients ingested peanuts once or twice a day, with the dose being gradually increased until the maintenance dose (795 mg of peanut protein) was reached. The scheduled up-dosing was adjusted according to the severity of allergic reactions. In the maintenance phase, patients ingested the peanuts without increasing the dose and with a gradual reduction in the ingestion frequency to once a week. Patients who did not have allergic reactions at this ingestion frequency for 4 weeks completely avoided peanuts for at least 2 weeks, followed by an OFC to confirm SU. In case allergic reactions occurred during the OFC for confirming SU, patients ingested peanuts on the same schedule as in the maintenance phase, followed by a repeat OFC. The build-up phase and maintenance phase could be achieved in the shortest period of 126 days and 266 days, respectively. Our OIT protocol could help achieve SU within as little as 280 days including the 2-week complete avoidance phase.

### 2.5. OFC protocols

OFCs were conducted based on the Japanese guidelines for FA [1]. The attending physician determined the challenge dose based on the patient’s history of allergic reactions or the specific IgE levels in reaction to peanuts or Ara h 2. The total challenge dose was administered in 2 fragmented doses comprising 1/3 and 2/3 portions of the total target dose at 40-minute intervals. The OFC was stopped and considered “positive” if the patient showed obvious objective symptoms.

### 2.6. Definitions of outcomes

SU was defined as a negative response to an OFC involving 795 mg of peanut protein after ≥2 weeks of complete avoidance. Patients who reached the maintenance phase were considered to have achieved peanut desensitization. Adverse events were determined based on the frequency of and treatment for allergic reactions, including adrenaline injections, during the build-up or maintenance phase.

### 2.7. Risk factors

We evaluated the following baseline risk factors for failure to achieve SU: age at OIT initiation, sex, history of anaphylaxis to peanuts, comorbid allergic diseases, total IgE and peanut-specific IgE levels at OIT initiation, and threshold dose. Participants were classified into high and low groups based on the median total IgE levels, peanut-specific IgE levels, and threshold dose. Potential risk factors were selected based on previous reports regarding factors related to successful OIT [16, 24, 25]. The relationship between the potential factors and failure to achieve SU was assessed using the log-rank test and Cox proportional hazards regression for univariate and multivariate analysis, respectively.

### 2.8. Adverse events

Parents monitored their children for ≥1 hour after home dosing and recorded the occurrence of any adverse reactions, the timing from dosing, and treatments administered in a study-provided diary. Adverse reactions were graded from grade 0 to 3 based on the modified Sampson classification [26]. Additionally, the parents’ diaries were reviewed at the hospital visit.

### 2.9. Immunological tests

The specific IgE levels in reaction to peanut and Ara h 2 were determined using Immuno CAP (Phadia, Thermo Fisher Scientific Diagnostics, Waltham, Massachusetts, MA, USA) at OIT initiation as well as after 12, 18, and 24 months of treatment.

### 2.10. Statistical analysis

The Kaplan–Meier curve was used for descriptive analyses of the duration until achievement of SU and desensitization. Data were collected until December 31, 2022. Patients were considered lost to follow-up if they dropped out of the OIT at the last contact or follow-up date. All patients were followed up from baseline until achievement of SU or censoring. Baseline was defined as the date of OIT initiation. The Kaplan–Meier curve was analyzed from the date of OIT initiation to the date of achievement of desensitization and SU. Hazard ratios and 95% confidence intervals (CI) for risk factors for failure to achieve SU were estimated using a Cox proportional hazards model. Within-group comparisons of the specific IgE levels in reaction to peanut and Ara h 2 were performed using the Wilcoxon signed-rank test. The Steel–Dwass test was used for multiple comparisons of the number of adverse events in the maintenance phase. Numerical values are expressed as number (%) or the median and interquartile range (IQR). Statistical significance was set at *P* < 0.05. All statistical analyses were performed using JMP Pro 15 (SAS Institute Inc. Cary, NC, USA).

## 3. Results

### 3.1. Demographic characteristics

**Figure 2.**
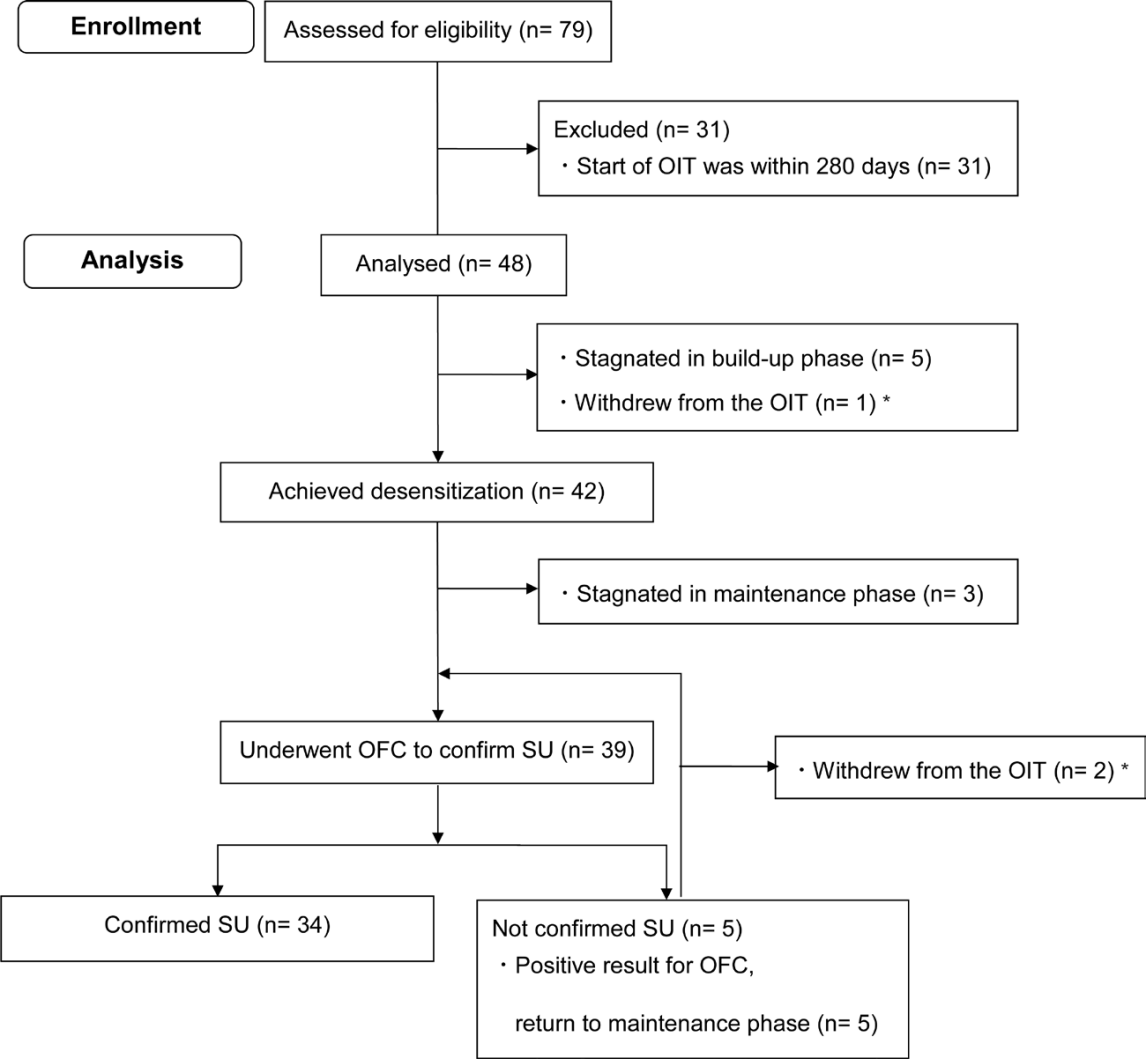
Consort diagram of patient selection to SU achievement. OFC, oral food challenge; OIT, oral immunotherapy; SU, sustained unresponsiveness. * Three patients withdrew from the OIT owing to the frequent occurrence of allergic reactions.

Among 79 patients with peanut allergy who underwent OIT during the study period, we excluded 31 patients who started OIT within 280 days. Finally, we enrolled 48 children (Fig. 2) in the study. The median observational period was 2.1 (IQR, 1.6–3.0) person-years (PY). At OIT initiation, the median age of the included children was 8 (7–10) years. Forty-one (85%) had a history of anaphylactic reactions to peanuts. The median total IgE level was 678 (IQR, 437–1,324) IU/mL. The median specific IgE level to peanut and Ara h 2 was 85.3 (IQR, 33.7–100) and 57.6 (IQR, 21.9–100) UA/mL, respectively (Table 1). There was no significant difference in background patients’ characteristics between the 48 patients who enrolled in the study and the 31 patients who were not enrolled (Supplementary Table 2 http://links.lww.com/PA9/A7).

**Table 1.** Characteristics at OIT initiation

	N = 48
Age at OIT initiation (years)	8 (7–10)
Male. Gender, n (%)	31 (65%)
Past history of anaphylaxis to peanut, n (%)	41 (85%)
Allergic complications	
Atopic dermatitis, n (%)	21 (44%)
Bronchial asthma, n (%)	26 (54%)
Allergic rhinitis, n (%)	19 (40%)
Total IgE level (I U/mL)*	678 (437–1,324)
Peanut specific IgE (UA/mL)*	85.3 (33.7–100)
Ara h 2 specific IgE (UAmL)*	57.6 (21.9–100)
Threshold of baseline OFC (mg)	26.5 (26.5–66.3)
OIT starting dose (mg)	13.3 (13.3–26.5)

Numerical values showed number (%) or the median and interquartile ranges (IQR).

When a child had obvious objective symptoms, we diagnosed as "positive" in oral food challenge.

IgE, immunoglobulin E; OFC, oral food challenge; OIT, oral immunotherapy.

* The level at OIT start.

At the initial OFC, the median threshold for eliciting allergic reaction at initial OFC was 26.5 mg of peanut protein among those who were OFC positive. Five children, who had previously provoked obvious allergic reaction to peanut, had a negative result for the initial OFC (Supplementary Table 3 http://links.lww.com/PA9/A8). Forty-two (88%) children achieved desensitization; The median period from the OIT start was 1.0 (0.7–1.4) years. Further, 39 of 42 children who achieved desensitization underwent OFC to confirm SU. The other 3 patients were in the maintenance phase. Of 39 children, 34 (71%) had confirmed SU: 26 (67%) in the first OFC and 8 (24%) in the second. The first OFC for SU was provided 229 (191–507) days from the confirmed desensitization. The median SU achievement period from OIT initiation was 2.1 (1.6–2.5) years. Three (6%) children withdrew from the OIT (1 in the build-up phase and 2 in the maintenance phase) owing to frequent provocation of allergic reactions (Fig. 2). The SU achievement rates did not significantly differ between patients with a positive and negative initial OFC (*P* = 0.63; Table 4). Kaplan–Meier analysis also showed that the difference in sustained unresponsiveness achievement rate did not significantly differ according to the initial OFC results (log-rank p=0.11; Supplementary Figure 2 http://links.lww.com/PA9/A4).

### 3.2. SU and desensitization

**Figure 3.**
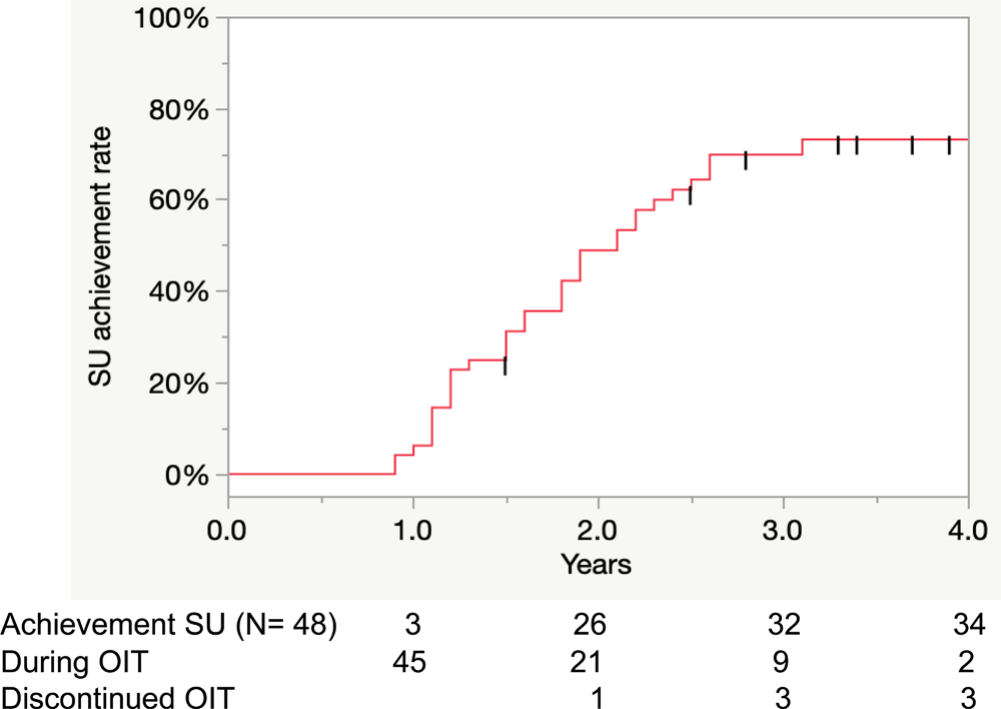
SU achievement rate. OIT, oral immunotherapy; SU, sustained unresponsiveness.

**Figure 4.**
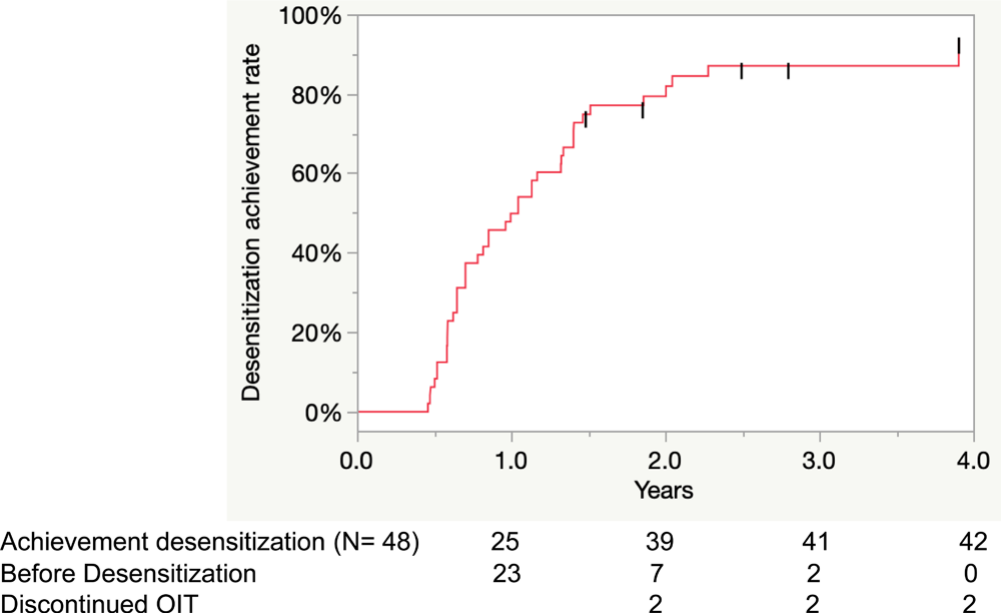
Desensitization achievement rate. OIT, oral immunotherapy.

The rates of SU and desensitization achievement gradually increased with a longer therapeutic period (Figs. 3,4). The rate of SU and desensitization achievement slowed down at 2.7 PY and 2.3 PY, respectively, after OIT initiation.

### 3.3. Adverse events

Of 48 children, 42 (88%) had at least 1 adverse event during the observational period. In total, 8,655 and 3,715 doses were administered during the build-up and maintenance phases, respectively. Compared with the build-up phase (159/8,655 [1.8%]), the maintenance phase (111/3,715 [3.0%]) had a significantly higher frequency of adverse events (*P* < 0.01). In the maintenance phase, there was no clear inverse trend between the rate of adverse events and the dosing frequency; adverse events in maintenance phase were 54/2,503 (2.2%) during the “once a day every day” phase, 31/608 (5.1%) during the “once a day every other day” phase, 21/387 (5.4%) during the “once a day twice a week” phase, and 5/217 (2.3%) during the “once a day once a week” phase, respectively. There was no significant difference in the frequencies of adverse events between phases (*P* = 0.39).

No cases of grade 3 symptoms were observed during the build-up or maintenance phase. Within the 8,655 doses administered in the build-up phase, 13 (0.2%) grade 2 symptoms were observed, with the most common being respiratory symptoms. Within the 3,715 doses administered in the maintenance phase, 23 (0.6%) grade 2 symptoms were observed, with the most common being skin symptoms. One patient received an intramuscular adrenaline injection owing to persistent cough (grade 2) after exercising following peanut ingestion during the maintenance phase (Tables 2,3).

**Table 2.** Adverse event during OIT in build-up phase (n = 48)

Adverse reactions	Total number of intakes of OIT	Total number of adverse reactions		
	8,655	159 (1.8%)		
Symptoms	Grade 0	Grade 1	Grade 2	Grade 3
Cardiovascular	–	–	0	0
Gastrointestinal	23	6	0	0
Respiratory	8	10	12	0
Skin	85	14	1	–
Treatment	Antihistamine	β2-inhalation	Adrenaline	Visiting hospital
	29	12	0	2

OIT, oral immunotherapy.

**Table 3.** Adverse event during OIT in maintenance phase (n = 42)

Adverse reactions	Total number of intakes of OIT	Total number of adverse reactions		
	3,715	111 (3.0%)		
Symptoms	Grade 0	Grade 1	Grade 2	Grade 3
Cardiovascular	–	–	1	0
Gastrointestinal	19	7	1	0
Respiratory	8	10	8	0
Skin	10	34	13	–
Treatment	Antihistamine	β2-inhalation	Adrenaline	Visiting hospital
	31	18	1	3

OIT, oral immunotherapy.

### 3.4. Risk factors for failure to achieve SU

Univariate and multivariate analyses revealed that having AD at initiation significantly decreased the risk of SU failure (hazard ratio, 0.30; 95% CI, 0.15–0.61; *P* < 0.01; adjusted hazard ratio, 0.18; 95% CI, 0.05–0.62; *P* < 0.01) (Table 4). No other risk factor was significantly associated with SU failure. Univariate analysis revealed that higher total IgE levels and higher threshold doses for eliciting allergic reaction at initial OFC decreased the risk of SU failure, but were not significant in the multivariate analysis.

**Table 4.** Risk factors for SU failure

	HR (95% CI)	*P* value	aHR* (95% CI) n = 48	*P* value
Age at OIT initiation	1.17 (0.58–2.38)	0.67	1.12 (0.91–1.20)	0.09
Male. Gender	0.89 (0.42–1.82)	0.85	2.37 (0.94–5.85)	0.07
Past history of anaphylaxis to peanut	0.92 (0.38–2.72)	0.87	1.50 (0.44–6.15)	0.54
Atopic dermatitis	0.30 (0.15–0.61)	<0.01	0.24 (0.09–0.57)	<0.01
Bronchial asthma	0.69 (0.35–1.37)	0.29	1.71 (0.76–3.93)	0.19
Total IgE (Log 10)	0.17 (0.09–0.45)	<0.01	1.50 (0.30–8.34)	0.63
Peanut specific IgE (Log 10)	2.93 (1.21–8.79)	0.02	1.66 (0.47–6.92)	0.44
Low threshold dose	3.92 (1.95–8.25)	<0.01	3.57 (1.37–9.71)	<0.01

Subjects were classified into high and low threshold dose groups based on low dose peanut OFC level (26.5 mg of peanut protein).

aHR, adjusted hazard ratio; 95% CI, confidence intervals; HR, hazard ratio; IgE, immunoglobulin E; OIT, oral immunotherapy; SU, sustained unresponsiveness.

* Adjusted for age of OIT initiation, gender, past history of anaphylaxis to peanut, atopic dermatitis, bronchial asthma, the level of total IgE, the level of specific IgE to Peanut, and the level of threshold dose.

### 3.5. Immunological tests

Twelve months after OIT initiation, the median specific IgE level in reaction to peanut and Ara h 2 significantly decreased to 49.6 (IQR, 21.3–76.4) UA/mL (−16.5%; 95% CI, −22.4% to −10.5%; *P* < 0.01) and 32.9 (IQR, 14.5–68.1) UA/mL (−15.8%; 95% CI, −20.7% to −11.0%; *P* < 0.01), respectively (Supplementary Fig. 1 http://links.lww.com/PA9/A3). The median peanut-specific IgE levels significantly decreased to 32.1 UA/mL at 18 months in 32 patients and 21.6 UA/mL at 24 months in 21 patients. The median Ara h 2-specific IgE levels decreased to 25.2 UA/mL at 18 months in 32 patients and to 19.8 UA/mL at 24 months in 21 patients.

## 4. Discussion

In this study, we investigated the optimal duration of OIT for achieving SU to peanuts and found that a longer period of OIT may increase the likelihood of achieving SU. However, the treatment efficacy can diminish with an increasing therapeutic period, especially if it exceeds 2.7 PY. Apart from the presence of AD, none of the other factors at OIT initiation were associated with SU failure. No grade 3 severe adverse events were observed during OIT.

Analysis of the association between the treatment period and SU achievement suggested reduced treatment efficacy when OIT was administered beyond the first 2.7 years. Since previous studies on OIT mainly focused on the efficacy within a predetermined building-up and maintenance treatment period of at most 1 to 2 years, there is little evidence to inform the choice of treatment options for patients with a poor response [13, 16, 18]. Peanut OIT greatly improves the QOL of patients, with SU or desensitization being achieved with fewer concerns regarding accidental exposures and severe allergic reactions as well as fewer limitations in dietary choices and social interactions [27, 28]. These findings point toward continued therapy for patients with poor responses. However, our findings suggest that the maintenance phase has a higher frequency of adverse events than the build-up phase, indicating that the risk of OIT-related allergic reactions persists throughout the treatment period [11]. Therefore, the risk of continuing peanut OIT beyond 2.7 years may outweigh the benefits in patients with a poor response. Given the low statistical power of our study, we should consider stopping OIT after 2.7 PY; however, this should be discussed with the patients.

The investigation of adverse events during OIT showed that patients were exposed to the mild adverse reactions throughout the therapeutic period and that adverse events were more frequent during the maintenance phase than during the build-up phase. These findings were consistent with those of a previous adverse event study of peanut OIT, which reported that allergic symptoms were induced even in the maintenance phase [25]. However, our OIT protocol, which gradually reduced the frequency of ingestion, may have increased the frequency of adverse events throughout the maintenance phase. We adopted this procedure because the abrupt cessation of allergen dosing may lead to increased severity of allergic reactions in the OFC to confirm SU. However, it is possible that the timing of dosing frequency reduction in our study was too rapid, resulting in the higher frequency of adverse events in the maintenance phase because there is a lack of knowledge about the optimal period to safely reduce the frequency of allergen intake after daily intake. Although the frequency of adverse events in this study did not differ between the dosing frequency steps, further investigation with a larger number of participants is warranted, as the occurrence of adverse events is 1 of the main reasons for the discontinuation of OIT [29].

Our findings showed that the presence of AD before OIT initiation significantly reduced the risk of SU failure. Direct comparison of the findings with the previous studies was difficult owing to differences in subject characteristics and OIT protocols, such as duration of therapy and target dosing. However, a study in children with severe peanut allergy showed a higher rate of concomitant AD in those who reached the targeted dosing (5,000 mg of peanut protein) than in those who discontinued OIT or reached the lower dose than the target. This difference in the rate in the previous study was not statistically significant but was consistent with our findings [18]. One possible explanation for why children with concomitant AD had more favorable outcomes in our study is that we provided appropriate treatment for AD as part of the general treatment during OIT. Treatment of AD improves skin barrier control, which not only reduces skin inflammation and improves AD disease expression, but also likely improves overall immune dysregulation and decreases Th2 initiation and effector responses [30]. In addition, the reduction of environmental peanut exposure owing to the improved skin barrier dysfunction may have contributed to allergy remission. The association between food sensitization through the skin and the development and remission of FA has been well-documented for wheat allergy induced by the use of hydrolyzed wheat protein soap [31]. In this type of wheat allergy, ~50% of patents resolved their wheat allergy by avoiding the skin exposure to the offending soap [32]. Given the strength of the association between AD and SU achievement in our study, future large-scale studies that include details regarding individual AD characteristics are warranted to validate this finding.

This study has several limitations. First, we included a small number of patients, especially beyond the 3-year observation period. The low statistical power may reduce the reliability of our findings regarding the long-term efficacy of the OIT. Compared to studies in Western countries, our study has a lower number of participants. The frequency of peanut consumption in Japan is not as high as that in Western countries, resulting in challenges in enrolling patients willing to participate in peanut OIT. Compared to previous Japanese studies on peanut OIT, this study had a higher number of participants [15]. Second, we did not evaluate the effect of tapering schedules and determined the definition of SU achievement. Specifically, the confirmation of SU differed in terms of the target dose and duration of complete avoidance period. Moreover, because the OIT protocol varies from study to study, the generalizability of our findings may be reduced. We applied a 2-week complete avoidance phase in accordance with a recent report [15]. A previous study on peanut OIT reported a decrease in the tolerated dose with prolonged avoidance [33]. Therefore, a 2-week complete avoidance phase may be insufficient for evaluating SU [16, 34-36]. However, most patients who enrolled in our study underwent OIT to prevent anaphylaxis due to accidental exposure. Therefore, if peanuts can be safely ingested after 2 weeks of complete avoidance, there is a reduced risk of anaphylaxis in daily life. Studies involving a longer period of complete avoidance or a higher target dose [16, 34-36] may report different findings regarding the SU achievement rate [16, 34, 35]. Third, the initial OFC dose differed across patients, which introduces a risk of bias since it was determined based on the medical history and at the discretion of the attending physician.

In conclusion, our findings demonstrated that a longer period of OIT may increase the likelihood of achieving SU. However, the rate of SU achievement slowed down after 2.7 PY from OIT initiation, indicating that for long-term OIT, we should consider discussing the continuation of OIT with patients, including changing the SU goal or stopping OIT.

## Conflicts of interest

The authors have no financial conflicts of interest.

## Author contributions

Kosei Yamashita, Toshinori Nakamura, and Takanori Imai designed the study. Kosei Yamashita, Aiko Honda., Yuki Okada, Mayu Maeda, and Toshinori Nakamura collected and analyzed data. Kosei Yamashita and Toshinori Nakamura wrote the manuscript; Toshinori Nakamura, Yuki Okada, Taro Kamiya, and Takanori Imai gave conceptual advice. All authors read and approved the final manuscript.

## Acknowledgements

We thank the children who participated in this study and their parents.

## Supplementary Material
