# Supplementary figures and images for: Optimal period for achieving sustained unresponsiveness in peanut oral immunotherapy
Source: Asia Pac Allergy. 2023 Sep 7;13(3):97–104. doi: 10.5415/apallergy.0000000000000110 (PMC10516315; doi:10.5415/apallergy.0000000000000110)

# Supplementary Figure 2: Difference in SU achievement rate between initial OFC results

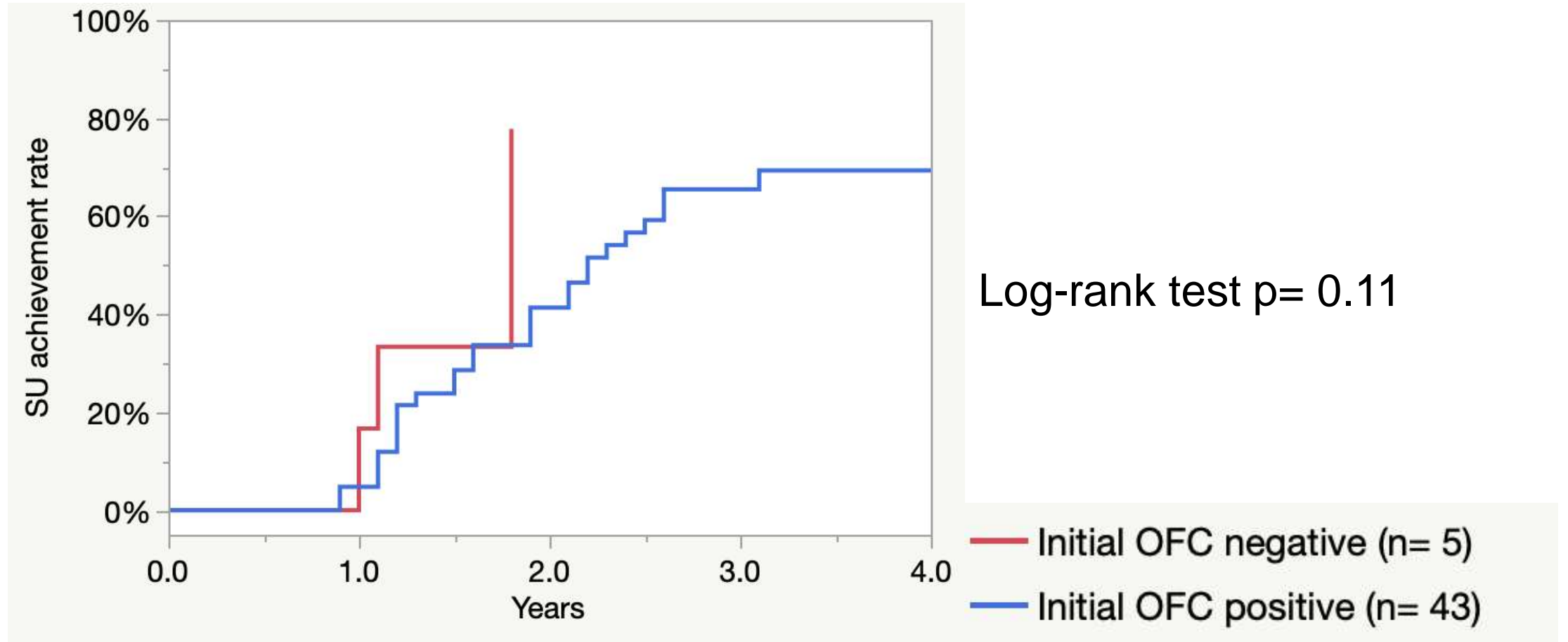

Supplement: Supplementary file 4 [file pa9-13-097-s004.pdf]

# Supplementary Figure 1: Change in peanut and Ara h 2 specific IgE level

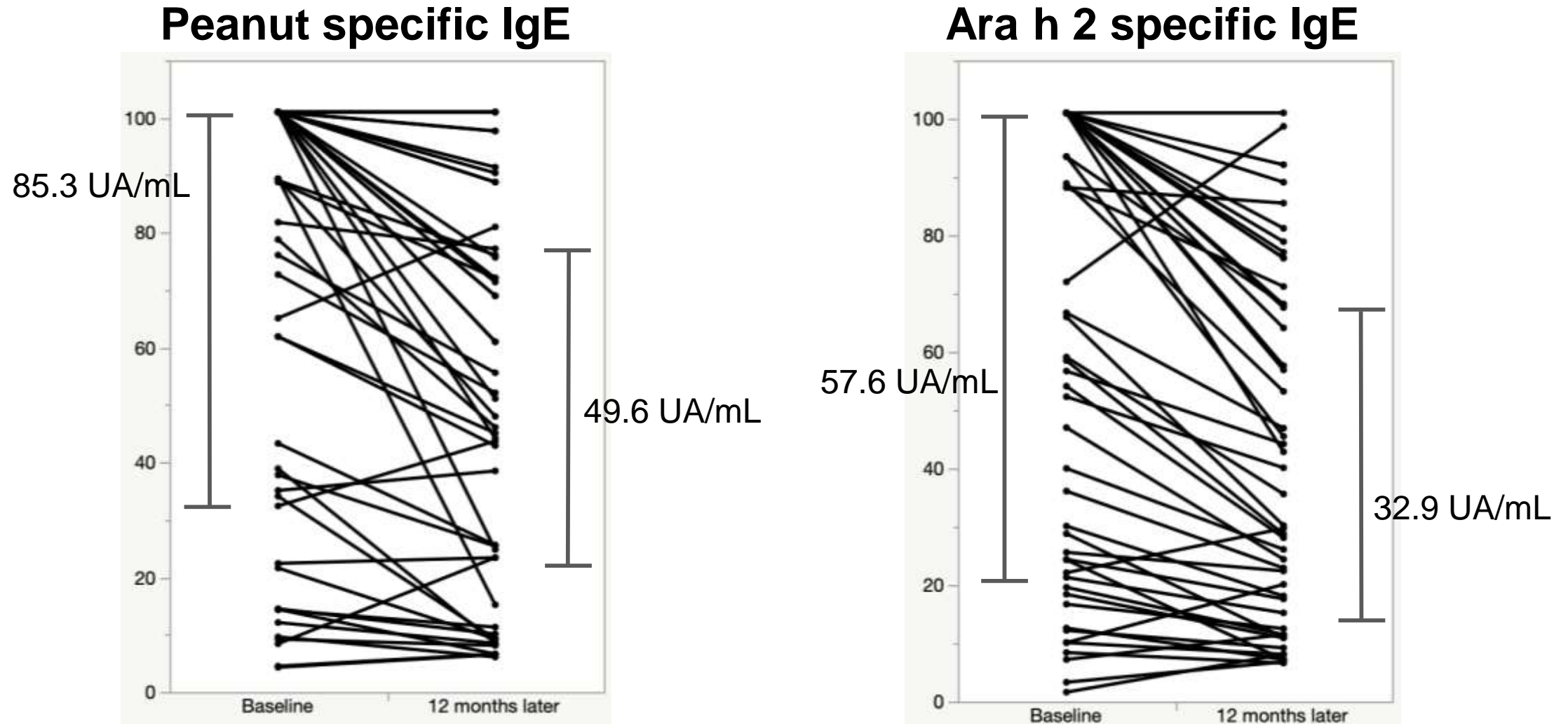

Supplement: Supplementary file 5 [file pa9-13-097-s005.pdf]
